# Traditional practices influencing the use of maternal health care services in Indonesia

**DOI:** 10.1371/journal.pone.0257032

**Published:** 2021-09-10

**Authors:** Ni Ketut Aryastami, Rofingatul Mubasyiroh

**Affiliations:** National Institute of Health Research and Development, Ministry of Health, Jakarta, Republic of Indonesia; Waikato Institute of Technology, NEW ZEALAND

## Abstract

**Background:**

Maternal Mortality Ratio (MMR) in Indonesia is still high, 305, compared to 240 deaths per 100,000 in South East Asian Region. The use of Traditional Birth Attendance (TBA) as a cascade for maternal health and delivery, suspected to be the pocket of the MMR problem. The study aimed to assess the influence of traditional practices on maternal health services in Indonesia.

**Methods:**

We used two data sets of national surveys for this secondary data analysis. The samples included 14,798 mothers whose final delivery was between January 2005 and August 2010. The dependent variables were utilization of maternal healthcare, including receiving antenatal care (ANC≥4), attended by skilled birth attendance (SBA), and having a facility-based delivery (FBD). The independent variables were the use of traditional practices, type of family structure, and TBA density. We run a Multivariate logistic regression for the analysis by controlling all the covariates.

**Results:**

Traditional practices and high TBA density have significantly inhibited the mother’s access to maternal health services. Mothers who completed antenatal care were 15.6% lost the cascade of facility-based delivery. The higher the TBA population, the lower cascade of the use of Maternal Health Services irrespective of the economic quintile. Mothers in villages with a high TBA density had significantly lower odds (AOR = 0.30; CI = 0.24–0.38; p<0.01) than mothers in towns with low TBA density. Moreover, mothers who lived in an extended family had positively significantly higher odds (AOR = 1.33, CI = 1.17–1.52; p<0.01) of using maternal health services.

**Discussion:**

Not all mothers who have received proper antenatal delivered the baby in health care facilities or preferred a traditional birth attendance instead. Traditional practices influenced the ideal utilization of maternal health care. Maternal health care utilization can be improved by community empowerment through the maternal health policy to easier mothers get delivery in a health care facility.

## Introduction

Indonesian maternal mortality ratio (MMR), according to the Intercencus Population Survey (SUPAS) 2015, was 305 per 100,000 live births [1]. It had been reducing from 359 in 2012; it is remained high compared to the neighboring countries with an average of 240 [2]. The three common delays that adversely affect maternal mortality are, first delay in family decision making due to mothers’ inability to recognize danger signs in pregnancy. The second and third delays are accessing care at a healthcare facility related to geographic barriers and problems with transportation; lastly, delay in receiving care because of the health service quality in healthcare facilities [3]. These three types of delays together caused 85.7 percent of maternal deaths. Maternal death is significantly associated with vaginal bleeding (59.7%), eclampsy (25.5%), depicted by a study in the Aceh province of Indonesia [4].

Limiting the role of TBAs in mother’s delivery to partnering with the SBA and assisting the mother to access the SBA and health care facilities is one of the Indonesian maternal health policies [5]. Later policy improvement was that delivery only possible in a health care facility instead of the mother’s home by SBA. The significant improvement of the policy is that mother’s delivery in health care facilities is free of charge [6,7]. MMR reduction is the principal purpose of the whole policy.

As an immense archipelago, Indonesia lies across the Equator and spans a distance equivalent to one-eighth of Earth’s circumference; composed of about 17,500 islands with more than 300 different ethnic groups and more than twice distinct languages bring about extraordinary diversity across the population. Traditional beliefs and practices are prevalent in Indonesia, similar to other Asian countries, and influence maternal and child health [8–10]. Several studies in Asia and Africa have used quantitative and qualitative approaches to identify traditional beliefs and practices for pregnant women, including dietary rules, personal hygiene, daily activities, staying apart from the family, taboos in informing about pregnancy, and prohibition of sexual intercourse [9,11–13]. Concerning delivery and postpartum practices, the belief, among others, the choice of delivery place, fear of exposure to hospital equipment, the practices, and rituals of cutting the umbilical cord placenta, throw the colostrum of the breastfeeding and hence the tradition of isolating the mother after childbirth [9,11,13–15].

Antenatal care (ANC) and delivery in health facilities have low access to maternal health services. It is associated with wealth index, ability to pay, mother’s knowledge and education, culture and belief, distance and transportation, or access to health facilities, including its capacity in the decision-making process [16–19]. With the use of skill birth attendance on delivery, a study found that the national health insurance called *JKN* for pregnant mothers has significantly increased professional birth attendance and primary health centers in Indonesia [18].

Indonesia’s varied cultures and ethnic group practices underlie health-seeking behavior for maternal and neonatal health (MNH) services, whether or not these influences are recognized [19]. These referred to the number of TBA in population, traditional practices, and family composition. A study in Uganda found that cultural factors inhibit mothers in using modern ANC. Beliefs and myths often force the mothers to give birth at home and implement the practice of traditional umbilical cord-cutting (UCC) [20].

Childbirth at home remains prevalent in rural areas in Indonesia. It is related to the presence of and preference for TBAs at the village level. Data from several small qualitative studies in Jakarta, Banten, and West Java found that TBA use was a significant socio-cultural barrier to MNH utilization [21]. The traditional beliefs underlying TBA preference were particularly pervasive among low-income families [22]. Research also showed that women tended to prefer TBAs because they believed it was easier to interact, have more experiences, are more accessible, and are more likely to encourage natural birth than midwives [23]. Many types and styles of services performed by TBAs could result in unexpected maternal and newborn complications. Such practices include *ngolesi* (to wet the vagina using coconut oil in the perception to ease the baby’s delivery). Other methods, called *kodok* (the TBA inserting a hand into the uterus to take out the mother’s placenta), *nyanda* (having the mother sit in a straight-legged position for hours, with a consequent risk of bleeding and swelling) [24].

Traditional UCC using bamboo knives—*sembilu*—or unsterilized razors or scissors is another traditional practice associated with maternal and newborn complications. Dayak’s ethnic group who mostly lives in Senggau, West Kalimantan, is widely practiced. Improper UCC may cause bleeding in the infant. Similarly, cutting the placenta using a bamboo knife may cause infection for the mother. Other risk factors associated with traditional UCC are mixed kitchen dust or coffee and a betel chewer’s saliva as an antiseptic, increasing the risk of maternal and newborn complications [25].

Evidence of the influence of family composition on maternal health services is very limited in Indonesia. Postnatal care’s increased use resulted from living in an extended family found in Central Java [26]. In other studies, familial support significantly increased ANC services using the Indonesian Demographic and Health Survey [14].

Studies found problems related to the unmet needs of family planning methods in many countries. Several cultural barriers exist, such as religious prohibitions, resistance from men, and misperceptions of the side effects of contraceptives [27–30]. This study aims to analyze traditional practices that influence comprehensive maternal health service use in Indonesia.

## Materials and methods

### Study design and participants

This study used cross-sectional data from the 2010 Indonesian Basic Health Research Study (*Riskesdas* 2010) and the 2008 Village Potential Survey (PODES). Although there are two newer *Riskesdas* datasets for 2013 and 2018, we used the *Riskesdas* 2010 dataset because it encompassed more variables related to maternal health than the latter. The later data (Riskesdas 2013 and 2018) have no variables to proximate the traditional practices such as UCC care and family planning use for traditional reasons. *Riskesdas* is a nationally representative survey, conducted every five years in all 33 provinces and 441 districts in Indonesia by the National Institute of Health Research and Development (NIHRD), Ministry of Health, and focuses on the measurement of health indicators mandated by the Millenium Development Goals or Sustainable Development Goals, such as reducing child mortality and improving maternal health.

The Ethics Committee of the NIHRD provided ethical clearance before the data collection. Because we conducted secondary data analysis, the prerequisite of ethics approval is not applicable. Before the data collection during the survey and interview, the enumerators raised inform consent to be agreed and signed up by the respondents.

*The* sampling technique was a two-stage stratified random sampling using neighborhood census blocks selected from each district/municipality in proportion to population size. The survey chooses 2,800 census blocks chosen randomly from all possible census blocks. In the second stage, 25 households were randomly selected from each census block, yielding 251,388 individuals from 70,000 homes. The study sample focuses on mothers who had a live birth in the five years before data collection (January 2005 to August 2010), for a total sample size of 14,798 mothers.

The 2008 PODES was used to generate a variable on TBA density per 1,000 population. PODES 2008 is also nationally representative and conducted every three years by the Central Bureau of Statistics [31]. PODES collected data on the following topics: availability of human resources, natural resources, infrastructure, public facilities, and economic and social facilities at the village level.

### Measurement of variables

#### Outcome variables

We examined the factors that influence maternal healthcare use—specifically, four outcome variables describing the maternal health continuum of care:

1. ANC use: Defined as the use of ANC services four times or more (ANC≥4) by a health professional (midwife or doctor, either at home or in a health facility)
2. SBA use for first contact: Defined as the direct use of health professionals for delivery.
3. ANC>4, SBA use for first and last contact: Defined as the use of ANC≥4 and the whole process of mother’s delivery attended by a health professional.
4. Use of continuum maternal health services: Defined as the use of ANC≥4 and the whole process of mother’s delivery by a health professional in addition to the health care facility.

#### Explanatory variables

We developed three variables to measure cultural constraints that may influence maternal healthcare use: (1) traditional practices, (2) family composition, and (3) TBA density. The first variable, “traditional practices,” refers to the mother’s conventional beliefs or behaviors and family/community. Traditional practices were proximate in a composite of (a) use of UCC care and (b) lack family planning use for traditional reasons [20,25]. We took a comparative approach to the impact of belief and tradition to strengthen our argument, as using a single variable may inadequately represent our analysis of the cultural implications. The traditional practices coded as "yes" if the mother used either way, and "no" for otherwise. Finally, for the analysis, we compare mothers who do not use family planning for legal reasons to all other mothers (including those who practice family planning). Family planning conceptually refers to either before or after delivery related to belief and religion; nonetheless, it contains family planning uses after delivery in this particular analysis.

The second variable captures the mother’s family composition, categorized as either nuclear or extended family [17,26,32]. A family consists of a husband, wife, and children, without any other relative defined nuclear family. Meanwhile, the definition of extended is a mother living with any other adults having blood relations such as parents, parents in law, grandparent, or adult siblings. The third variable captures the availability of TBAs at the village level. Evidence shows that the more TBA in the community, the less likely the mother is to use modern MNH-seeking practices [21,23]. We measure TBA density as the number of TBA per 1,000 population. We categorized the TBA density into five quintiles from Q1 (low) to Q5 (highest).

#### Covariates

Our analysis controlled for demographic determinants (mother’s age, education, parity, residence type, and region based on five groups of islands). We also held household socio-economic status, i.e., average expenditure per capita per month divided into five quintiles, from Q1 (lowest or spending less than IDR 190,000 per month) to Q5 (richest or spending more than IDR 1,500,000 per month.). (S1 File_list of variables).

### Statistical analysis

Descriptive statistics used to describe the sample characteristics (Table 1). Categorical variables represent frequencies and their associated percentages. The proportion outcome of the dependent variables of ANC≥4, SBA, FBD, and all maternal continuum services based on (cross ordered groups) the independent variables of cultural barriers and mother’s characteristics examined using the Cuzick’s test for trend and Z-score (Table 2). Finally, we used multivariate logistic regression to quantify the relationship between potential explanatory variables for maternal health outcomes adjusted by the covariate (Table 3). The analysis was conducted by STATA version 14.0, considering the corresponding *Riskesda*s weights, strata, primary sampling unit accordingly to the *Riskesda*s survey design, as published elsewhere [33,34]. Plausible covariates for maternal care utilization were the length of education, age, the birth of the first child, socio-economic status, urban-rural location, and region by island group divided by the big islands.

**Table 1:** Sample characteristics and utilization of maternal health services.

Variables	N	%
Mother’s age at birth		
*15–24 years old*	3,962	26.6
*25–34 years old*	7,914	53.5
*35–49 years old*	2,922	19.9
Mother’s education		
*Primary school or less*	6,482	43.1
*Junior high school*	3,267	22.3
*Senior high school or more*	5,049	34.6
Parity		
*1*	4,162	28.6
*2*	4,904	33.3
*3*	2,892	19.5
*4+*	2,840	18.7
Household socio-economic status, by quintile.		
*Poorest*	3,541	23.9
*Poor*	3,272	22.3
*Middle*	3,055	20.5
*Rich*	2,792	18.7
*Richest*	2,138	14.6
Residence type		
*Rural*	7,425	48.7
*Urban*	7,373	51.3
Region		
*Java-Bali*	7,097	53.0
*Sumatra*	3,724	24.2
*Kalimantan*	1,202	7.1
*Sulawesi*	1,352	7.7
*Eastern Indonesia*	1,423	8.0
Traditional practices		
*No*	12,521	85.3
*Yes*	2,277	14.7
TBA density		
*Low*	3,541	24.2
*Few*	2,372	17.2
*Moderate*	2,964	20.2
*High*	2,968	19.9
*Highest*	2,953	18.5
Family composition		
*Nuclear*	12,673	85.9
*Extended*	2,125	14.1
Number of ANC visits		
0	1,076	6.6
1–3	2,267	14.6
4+	11,455	78.7
SBA first contact	10,796	74.2
SBA last contact	11,703	80.3
FBD	7,817	54.9
ANC≥4 and SBA first and last contact	9,190	63.6
ANC≥4, SBA first and last contact, and FBD	6,792	48.0

**Table 2:** Traditional practices and percent of maternal health services utilization.

Variables	ANC≥4		SBA first contact		ANC≥4, SBA first and last contact		All maternal continuum services	
	%	p-value	%	p-value	%	p-value	%	p-value
Traditional practices		<0.001		<0.001		<0.001		<0.001
*No*	82.1		79.0		68.1		51.6	
*Yes*	59.0		46.5		37.9		27.3	
TBA density		<0.001		<0.001		<0.001		<0.001
*Low*	85.1		88.9		78.6		65.2	
*Few*	89.9		87.6		80.6		72.5	
*Moderate*	82.2		77.6		66.2		48.1	
*High*	76.0		64.4		53.4		34.5	
*Highest*	58.9		49.3		36.5		17.2	
Family composition		<0.001		0.003		0.002		<0.001
*Nuclear*	78.2		73.8		63.1		47.4	
*Extended*	81.6		76.9		66.6		51.8	
Mother’s age at birth		0.486		<0.001		0.002		<0.001
*15–24*	76.3		69.4		58.7		42.6	
*25–34*	80.9		76.9		67.1		51.2	
*35–49*	75.8		73.3		60.9		46.7	
Mother’s education		<0.001		<0.001		<0.001		<0.001
*Primary school or less*	67.3		57.5		44.9		28.7	
*Junior high school*	81.7		79.0		67.8		49.2	
*Senior high school or more*	90.9		91.9		84.3		71.4	
Parity		<0.001		<0.001		<0.001		<0.001
*1*	84.0		79.0		70.2		55.2	
*2*	82.7		78.4		69.1		53.1	
*3*	77.1		73.1		62.1		46.4	
*4+*	65.0		60.4		45.6		29.7	
Household socio-economic status		<0.001		<0.001		<0.001		<0.001
*Poorest*	63.7		57.3		42.8		27.6	
*Poor*	75.9		68.5		56.9		39.4	
*Middle*	81.3		77.9		66.9		49.2	
*Rich*	87.4		84.8		77.0		61.6	
*Richest*	92.6		91.9		86.5		75.8	
Residence type		<0.001		<0.001		<0.001		<0.001
*Rural*	68.5		61.8		48.6		28.2	
*Urban*	88.3		86.0		77.9		66.8	
Region		<0.001		<0.001		<0.001		<0.001
Java-Bali	87.1		78.6		72.2		60.9	
Sumatera	70.7		79.4		61.1		40.0	
Kalimantan	69.4		67.4		54.1		31.9	
Sulawesi	64.2		54.9		40.7		22.7	
Eastern Indonesia	68.8		53.9		45.2		25.6	
Total	78.7		74.2		63.6		48.0	

**Table 3:** Multivariate logistic regression of traditional practices and continuum of maternal health care utilization.

Variables	ANC≥4			SBA first contact			ANC≥4, SBA first and last contact			All maternal continuum services		
	AOR	p-value	(95% CI)	AOR	p-value	(95% CI)	AOR	p-value	(95% CI)	AOR	p-value	(95% CI)
Traditional practices												
*No (ref)*	1.00			1.00			1.00			1.00		
*Yes*	0.47	<0.001	(0.41–0.54)	0.27	<0.001	(0.24–0.31)	0.38	<0.001	(0.33–0.43)	0.50	<0.001	(0.43–0.58)
TBA density												
*Low (ref)*	1.00			1.00			1.00			1.00		
*Few*	1.04	0.664	(0.80–1.30)	0.68	0.020	(0.49–0.92)	0.81	0.099	(0.64–1.02)	0.94	0.629	(0.77–1.17)
*Moderate*	0.85	0.195	(0.68–1.04)	0.53	<0.001	(0.41–0.68)	0.60	<0.001	(0.49–0.73)	0.54	<0.001	(0.45–0.65)
*High*	0.81	0.067	(0.64–1.00)	0.38	<0.001	(0.29–0.48)	0.49	<0.001	(0.40–0.59)	0.44	<0.001	(0.36–0.54)
*Highest*	0.55	<0.001	(0.44–0.70)	0.26	<0.001	(0.20–0.34)	0.35	<0.001	(0.28–0.44)	0.30	<0.001	(0.24–0.38)
Family composition												
*Nuclear (ref)*	1.00			1.00			1.00			1.00		
*Extended*	1.28	0.003	(1.10–1.51)	1.34	<0.001	(1.15–1.55)	1.27	<0.001	(1.12–1.45)	1.33	<0.001	(1.17–1.52)
Mother’s age at birth												
*15–24 years old (ref)*	1.00			1.00			1.00			1.00		
*25–34 years old*	1.61	<0.001	(1.40–1.85)	1.78	<0.001	(1.55–2.04)	1.73	<0.001	(1.52–1.97)	1.62	<0.001	(1.43–1.84)
*35–49 years old*	1.62	<0.001	(1.33–1.98)	2.28	<0.001	(1.90–2.77)	1.97	<0.001	(1.65–2.35)	2.03	<0.001	(1.71–2.40)
Mother’s education												
*Primary school or less (ref)*	1.00			1.00			1.00			1.00		
*Junior high school*	1.69	<0.001	(1.47–1.94)	2.08	<0.001	(1.83–2.36)	1.99	<0.001	(1.76–2.24)	1.87	<0.001	(1.66–2.11)
*Senior high school or more*	2.61	<0.001	(2.26–3.01)	4.06	<0.001	(3.45–4.77)	3.35	<0.001	(2.95–3.80)	3.23	<0.001	(2.86–3.65)
Parity												
*1 (ref)*	1.00			1.00			1.00			1.00		
*2*	0.84	0.025	(0.72–0.98)	0.88	0.100	(0.76–1.02)	0.87	0.042	(0.76–0.99)	0.84	0.012	(0.74–0.96)
*3*	0.64	<0.001	(0.54–0.76)	0.65	<0.001	(0.54–0.78)	0.66	<0.001	(0.56–0.78)	0.67	<0.001	(0.57–0.78)
*4+*	0.49	<0.001	(0.40–0.60)	0.48	<0.001	(0.39–0.59)	0.46	<0.001	(0.38–0.55)	0.44	<0.001	(0.36–0.52)
Household socio-economic status												
*Poorest (ref)*	1.00			1.00			1.00			1.00		
*Poor*	1.45	<0.001	(1.26–1.66)	1.14	0.062	(0.99–1.32)	1.33	<0.001	(1.17–1.51)	1.25	0.001	(1.09–1.42)
*Middle*	1.68	<0.001	(1.44–1.97)	1.47	<0.001	(1.26–1.72)	1.64	<0.001	(1.43–1.89)	1.50	<0.001	(1.30–1.73)
*Rich*	2.12	<0.001	(1.77–2.55)	1.64	<0.001	(1.38–1.96)	2.06	<0.001	(1.76–2.41)	1.89	<0.001	(1.62–2.21)
*Richest*	2.88	<0.001	(2.28–3.63)	2.50	<0.001	(1.96–3.20)	2.97	<0.001	(2.44–3.62)	2.83	<0.001	(2.37–3.38)
Residence type												
*Rural (ref)*	1.00			1.00			1.00			1.00		
*Urban*	1.40	<0.001	(1.20–1.63)	1.51	<0.001	(1.28–1.8)	1.43	<0.001	(1.24–1.64)	1.85	<0.001	(1.62–2.11)
Region												
*Java-Bali (ref)*	1.00			1.00			1.00			1.00		
*Sumatra*	0.40	<0.001	(0.34–0.47)	1.52	<0.001	(1.26–1.84)	0.69	<0.001	(0.59–0.80)	0.45	<0.001	(0.39–0.52)
*Kalimantan*	0.42	<0.001	(0.34–0.52)	0.94	0.614	(0.72–1.21)	0.58	<0.001	(0.47–0.70)	0.32	<0.001	(0.25–0.41)
*Sulawesi*	0.42	<0.001	(0.33–0.53)	0.57	<0.001	(0.45–0.73)	0.39	<0.001	(0.32–0.49)	0.27	<0.001	(0.22–0.34)
*Eastern Indonesia*	0.66	0.001	(0.51–0.84)	0.70	0.003	(0.56–0.89)	0.64	<0.001	(0.51–0.80)	0.41	<0.001	(0.32–0.53)

## Results

### Sample characteristics

This analysis involved 14,798 mothers. Table 1 showed the sample’s characteristics. The majority of mothers (85.3%) reported non-traditional cultural practices, and (85.9%) lived in a nuclear family structure. Most mothers were ages 25–34 years at the time of their recent live birth (53.5%) with primary school education or less (43.1%); A significant proportion of low socio-economic quintiles of poor and abysmal (46.2%). The parity was second birth (33.3%), followed by first birth (28.6%). Mother’s residential distributed equally in urban and rural sites but mainly resided in the Java-Bali and Sumatra regions (S2 File_Table Analysis by region).

#### The proportion of mothers utilize maternal health services

Table 1 also depicted the use of maternal health services by mothers. The majority of mothers (78.7%) had antenatal visits at least four. The percentage of maternal care for delivery attendance declined to 74.2% for first delivery contact with a health professional (SBA). Nonetheless, the last SBA’s percentage increasing (80.3%) for the previous reference ended up with only 54.9% of mothers delivered at the health facilities.

A total of 63.6% of mothers continually utilized the maternal health services of ANC≥4 and SBA for their first and last delivery contact. The proportion was getting more minor (48%) for the completed or ideal continuum services for those using health facilities in maternal delivery (FBD).

Our bivariate analysis (Table 2) showed the proportion differences of the dependent variables (ANC≥4, SBA, FBD, and all maternal continuum services) based on cross ordered groups of the explanatory variables (cultural barriers and mother’s characteristics). All the dependent variables were significantly higher among mothers who did not adopt the traditional practice. The table also depicted the outcome distribution of the cascade of care according to its characteristics. The proxy of traditional practice seemed consistent and was lower in all maternal health care utilization. The ratio of TBAs was also constant; the higher the TBA ratio, the lower the proportion of mothers accessing proper ANC. The pattern cascade of care goes down for ideal service use; ANC≥4; SBA first contact; SBA first and last contact; and use of complete services (ANC≥4, SBA first and last contact, and FBD). The critical outcome of traditional practices’ influence is related to family patterns; mothers who lived in an extended family were more likely to continue using maternal health services. The plausible covariates for increasing the use of maternal care were better education (i.e., a higher level of mother’s knowledge), optimal age (25–34), parity, most elevated socio-economic status, urban location, and region (Java-Bali).

### Influence of traditional practices on maternal health utilization

Table 3 depicted the logistic regression results of traditional practices on the continuum of maternal healthcare services utilization. We found that conventional methods significantly influenced maternal healthcare utilization after controlling for demographic and socio-economic characteristics. Mothers who adopted traditional practices experienced substantially lower odds of using all maternal care services than their counterparts; AOR = 0.47 for ANC ≥4 and AOR = 0.27 for seeking SBA for first delivery contact (*p*<0.01). Mothers living in an extended family experienced a 1.3 times higher chance of receiving comprehensive maternal care than women living in a nuclear family (*p*<0.01).

Traditional practices have more than doubled the odds that a mother would not use maternal health services. As shown in Table 3, the adjusted odds ratio of ANC≥4 was 0.47, first contact with SBA (AOR = 0.27); ANC≥4 and skilled birth for the first and last contact (AOR = 0.38); and finally, use of excellent care of ANC≥4 times, SBA first contact, and FBD (AOR = 0.5).

Finally, the results showed that higher TBA density in the village significantly decreases the mother’s odds of the cascade of maternal health services. For example, mothers in villages with the highest TBA-to-population ratios having lower odds of receiving the complete maternal health continuum of services (AOR = 0.30) compared to mothers residing in villages with the lowest TBA density ratios (*p*<0.01). It is important to note that these relationships’ strength, significance, and direction hold when analyzing rural/urban residence effects. This association pattern was consistently significant for mothers in the Java-Bali islands and was conversely associated with the extended family structure (see Table 1 in the Appendix).

This analysis’s demographic and socio-economic characteristics indicated that both of these variables were significant predictors of maternal health services utilization. Mothers who reported completed high school or higher were more likely to use maternal health services than those whose education was low. Mothers in the richest socio-economic quintile experienced 2.8 times higher odds of using the completed cascade of maternal health services. Comparing the level of quintile, those who were at the highest quintile have significantly almost three-time better access to maternal services such as ANC≥4 (AOR = 2,9), SBA for first, and last delivery contact (AOR = 2,9) than the lowest quintile (*p*<0.01).

## Discussion

As expected, this study’s findings confirmed that various factors associated with traditional practices influenced the use of maternal health services. Mothers who adhered to conventional methods were less likely to use maternal health services compared to their counterparts. Our findings align with those presented by a study in Mexico where cultural barriers related to family planning were attributable to religious reasons, prohibition by husbands, and fear of side effects. Moreover, this study also mentioned that gender roles and religious objections acted intergenerationally to influence the refusal to use modern contraception methods. Besides, low education levels resulted in a lack of information and misconceptions about the long-term fertility risks due to hormonal exposure from modern methods [27,28].

The existence of the TBA density becomes a challenge for the health system. On the one hand, the Ministry of Health is trying to eliminate the number of TBA; on the other hand, the community’s preference keeps the TBA socially available. The adjusted policy has been implemented in some communities to hinder the power of TBA, by midwife-TBA partnership p. The role of TBA is to advise, encourage, and assist the mother by visiting a midwife to assist the SBA during the mother’s delivery. The TBA’s additional and more inclusive services to the mother are doing body massage to the pregnant mother, and after delivery, conduct newborn ceremonial, etcetera [5]. The TBA assists the midwife and facilitates the pregnant mother to ensure the health personnel midwife or doctor helps the mother’s delivery [5]. A study in Parung (West Java) supports our results mentioning that 45% of mothers deliver with TBA in addition to the firmer traditional belief scores of mothers [35].

Our results also indicate that family structure is an essential factor influencing mothers’ use of ANC≥4, SBA, and FBD. Mothers who live in an extended family more likely to use health facility is quite controversial with the previous era in Indonesia. In the old fashion, extended family has more intervene pregnant mother in encouraging a mother to find a traditional birth attendance for delivery. A survey in India in 1998–1999 supports this trend saying that a joining mother in an extended family prefers to use traditional care for ANC and delivery [36]. The extended family structure has a positive influence on the use of MNH services as a whole. These findings are consistent with a study in central Java that showed that the extended family is significantly associated with the help of antenatal and postnatal care [14,26]. Our findings are also consistent with the United States-based study, which found a significant influence on MNH utilization for mothers within an extended family comprising other adult members or co-parents [37].

The barriers to visit health facilities for mothers who live in a nuclear family seen from the cascade of access, which may occur due to the couple’s low education and lack of access to information. Patriarchal culture is among other brings about mother has less power in some communities [38]. In addition, a couple’s neglected risk of pregnancy is something familiar for a married couple; nevertheless, lesser evidence would happen in the urban areas as access and information related matter is easier to be cached up [39–41]. Our results, consistent with a study from Madhya Pradesh, India, showed that women living in an extended family that had good family relationships were more likely to receive antenatal services. [32]; nevertheless, after controlling for sociodemographic characteristics, women living in extended households were less likely to receive either ANC services or an FBD [36]. Qualitative studies in Egypt, Nepal, and China have also been inconclusive [42–44].

This study also found that TBAs are also associated with decreased utilization of the entire cascade of maternal health services. Mothers who live in an area with a high prevalence of TBAs were less likely to use maternal health services. In the last contact, which was higher than the first contact, SBA may evidence in mothers at first contact attended by TBA somehow having difficulties and then referred to the SBA. In general, mothers may view the TBAs as being familiar and of general competence. Indeed, other studies in Indonesia have confirmed that TBAs were assisting mothers ultimately throughout the maternal care provision continuously from pregnancy up to delivery and postnatal care hence up to 40 days after birth [45].

Some mothers who received ANC and contacted SBA do not deliver in a health facility were somehow less likely due to lack of the health system factors such as cost, distance or quality of care, but culture and preference. Mothers feel safer having the delivery close to the family and attended by the traditional birth attendance. In some ethnic groups in Indonesia, a TBA, known as a *dukun*, is considered someone with supernatural power without formal learning gained through cultural traditions [46]. Moreover, the TBA mothers received mantras, and herbs believed in making the delivery process more accessible and more comfortable [23]. A study in the Bogor district found that using a TBA for delivery was inexpensive and convenient because TBAs were perceived to be friendlier and more patient regarding the mother’s condition [45]. Contrary, a study in Cirebon, West Java, found some mothers preferred to deliver with a TBA even though they paid more than SBA [47].

Mother’s age and educational attainment influenced the use of maternal health care. Study results showed that the older and better educated the mother, the greater the odds of using maternal health services. These findings are consistent with those from a study in a Midwestern city in the United States, where years of education are significantly associated with the use of adequate prenatal care [48]. Conversely, a study in South Jakarta found that mothers’ age, education, occupation, and the number of pregnancies did not influence antenatal care seeking appropriately [49]. This contradiction may be due to the different studies’ criteria for choosing their subjects and the socioeconomics background; the former chose low-income women in a developed country. The latter chose women in middle-income countries with free antenatal services provided by the government. Nevertheless, these free services are not necessarily inexpensive, as geographical access and transportation barriers remain. As a large country with great socio-economic diversity, the gaps between poor and rich remain very wide, as was found by a study conducted in three districts in West Java [14].

Urban and rural residence and region were two demographic variables that signify the difference in maternal health utilization. In Indonesia, economic development has occurred more extensively in the western part of the country, such as in Java-Bali, compared to Nusa Tenggara, Maluku, and Papua in the eastern. Similarly, the distribution of health care facilities is more remarkable in urban areas and western Indonesia. The availability and consistent distribution of health facilities made mother’s in the urban population access more health facilities. In such conditions, access to a health facility depends not only on free service provision and facilities distribution but also on distance, and transportation availability, especially in a remote location. A study in West Java showed that distances from health facilities and poor road conditions constrained mothers’ access to antenatal care, particularly for those living in remote areas [17,50].

This study used *Riskesdas* 2010 data to analyze quantitative evidence on cultural factors or traditional practices on maternal health services in Indonesia. One limitation is that Riskesdas 2010 is an older dataset. However, it provides data that allowed us to analyze the influence of traditional practices at a nationally representative level compare to the newer data of *Riskesdas* 2013 and 2018. As cross-sectional data, this analysis can measure only association, not causation; however, the findings indicated that traditional beliefs and cultural issues are associated with maternal health in Indonesia.

## Conclusion

Traditional practices significantly influenced maternal care’s ideal utilization as a cascade from antenatal care to delivery in a health care facility in Indonesia after controlling for demographic and socio-economic characteristics. Some mothers access incomplete maternal health services from pregnancy to delivery, as recommended by the program. We concluded that conventional practices in utilizing maternal health services in Indonesia present a problem that may lead to a high prevalence of maternal mortality. Besides, as mentioned by other studies, delivery costs may also evident not associated with, but traditional practices and high TBA density significantly influence mother access to maternal health utilization. Our study lost some mothers that completed antenatal care the cascaded delivery in health facilities.

Higher TBA density in the village significantly decreases the mother’s odds of all maternal health services. It is important to note that these relationship’s strength, significance, and direction of these relationships hold when analyzing rural/urban residence effects. The association of mother’s education and socio-economic status was consistently significant for mothers in the Java-Bali islands and was conversely associated with the extended family structure. Immense family structures frequently exist in Sulawesi and Eastern Indonesia. At the same time, Sulawesi and Java-Bali have the firmest traditional beliefs and practices regarding contraceptive use as a family planning method.

Trust and preference of mothers are un-avoided when the maternal health programs and policy have improved from delivery by a health professional to health professional plus at the health care facilities. This study recommends that community empowerment involving local community leaders and spiritual channeling enable mothers to utilize health care facilities for pregnancy check and delivery concerning location and ethnicity. Improving knowledge about the risks of pregnancy for almost married couples is another option that possibly is developed and delivered through the civil registration system. When the couple register to be married, they are obliged to prepare for a healthy pregnancy. Further research that uses more comprehensive measures of traditional beliefs and practices still be needed to enhance the advocacy in escalating the reduction of maternal mortality in Indonesia.

## Supporting information

S1 File List of variables. (PDF)

S2 File Table analysis by region. (PDF)

## Abbreviations

ANC: antenatal care
FBD: facility-based delivery
IDR: Indonesia Rupiah
JKN: *Jaminan Kesehatan Nasional*, or national health insurance
MMR: Maternal mortality ratio
MNH: maternal, neonatal health
NIHRD: National Institute of Health Research and Development
PODES: *Potensi Desa* (Village Potential Survey)
SBA: skilled birth attendant
SUPAS: Intercencus Population Survey
TBA: traditional birth attendant
UCC: umbilical cord-cutting
