# Supplementary material for: Traditional practices influencing the use of maternal health care services in Indonesia
Source: PLoS One. 2021 Sep 10;16(9):e0257032. doi: 10.1371/journal.pone.0257032 (PMC8432883; doi:10.1371/journal.pone.0257032)
Supplement: S1 File — (PDF) [file pone.0257032.s001.pdf]

List of variables for Analysis

Title: Traditional Practices Influencing the Use of Maternal Health Care Services in Indonesia

NO	VARIABLE LABEL	DATA SET	INFORMATION NOTE
1.	B1R1	Riskesdas 2010	Province
2.	B1R5	Riskesdas 2010	Urban/rural location
3.	B4K3	Riskesdas 2010	Type of family where mother staying with (Nuclear/extended family)
4.	B4K8	Riskesdas 2010	Mother's education
5.	DB11	Riskesdas 2010	Number of children/parity
6.	DC06	Riskesdas 2010	Reasons for not using family planning method
7.	DD12	Riskesdas 2010	Place for seeking antenatal care during the last pregnancy
8.	DD13	Riskesdas 2010	Antenatal care was given by whom: traditional healer (*dukun*) or health personnel (midwife)
9.	DD16	Riskesdas 2010	The frequency for checking of antenatal care along the last pregnancy
10.	DD18	Riskesdas 2010	Frequency of prenatal care by trimester of pregnancy
11.	DD02B	Riskesdas 2010	Age of mother at the last delivery
12.	EA03AA	Riskesdas 2010	The first birth attendance for the last delivery
13.	EA03AB	Riskesdas 2010	The last (final) birth attendance for the last delivery
14.	EA03B	Riskesdas 2010	Place of delivery
15.	EA07	Riskesdas 2010	Any medicine or herbal apply for umbilical care of the newborn
16.	Household socioeconomic status	Riskesdas 2010	Composite variables of household expenditures
17.	R606e	Podes 2008	Number of traditional birth attendance living at the neighborhood of village or municipality
