# Supplementary material for: Traditional practices influencing the use of maternal health care services in Indonesia
Source: PLoS One. 2021 Sep 10;16(9):e0257032. doi: 10.1371/journal.pone.0257032 (PMC8432883; doi:10.1371/journal.pone.0257032)
Supplement: S2 File — (PDF) [file pone.0257032.s002.pdf]

Appendix

Table 1. Logistic regression of traditional practices and cascade of maternal health services utilization by regions

Explanatory Variables	ANC ≥4		SBA first contact		ANC ≥4, SBA first and last contact		All maternal continuum services	
	OR	p	OR	p	OR	p	OR	p
**REGION: JAVA - BALI**								
Family structure (Ref: Nuclear)								
Extended	1.27	0.049	1.47	<0.001	1.32	0.003	1.49	<0.001
Composite Cultural barrier variable (Ref: Modern)								
Traditional	0.44	<0.001	0.36	<0.001	0.46	<0.001	0.60	<0.001
Density of TBA (Ref: Q1 - lowest)								
Q2 - low	0.76	0.070	0.57	0.003	0.63	0.002	0.71	0.013
Q3 - moderate	0.56	<0.001	0.35	<0.001	0.37	<0.001	0.36	<0.001
Q4 - high	0.54	<0.001	0.28	<0.001	0.33	<0.001	0.32	<0.001
Q5 - highest	0.31	<0.001	0.20	<0.001	0.20	<0.001	0.18	<0.001
Mother's age at birth (Ref: 15-24 years)								
25-34	1.66	<0.001	1.66	<0.001	1.68	<0.001	1.54	<0.001
35-49	1.75	<0.001	2.31	<0.001	2.00	<0.001	2.06	<0.001
Mother's education (Ref: Primary school or less)								
Junior high school	1.95	<0.001	2.18	<0.001	2.22	<0.001	2.09	<0.001
Senior high school or more	3.72	<0.001	4.65	<0.001	4.32	<0.001	4.33	<0.001
Parity (Ref: 1)								
2	0.99	0.631	0.93	0.349	0.95	0.388	0.99	0.634
3	0.64	0.002	0.64	0.001	0.65	<0.001	0.78	0.022
≥4	0.38	<0.001	0.39	<0.001	0.36	<0.001	0.41	<0.001
Economic state (Ref: Poorest (Q1))								
Poor (Q2)	1.58	<0.001	1.27	0.006	1.37	<0.001	1.27	0.002
Middle (Q3)	1.72	<0.001	1.58	<0.001	1.59	<0.001	1.46	<0.001
Rich (Q4)	2.14	<0.001	1.75	<0.001	1.94	<0.001	1.79	<0.001
Richest (Q5)	2.64	<0.001	2.69	<0.001	2.77	<0.001	2.77	<0.001
Residence type (Ref: Rural)								
Urban	1.40	0.003	1.14	0.167	1.15	0.101	1.54	<0.001
**REGION: SUMATRA**								
Family structure (Ref: Nuclear)								
Extended	1.56	0.005	0.98	0.626	1.25	0.098	1.17	0.176
Composite Cultural barrier variable (Ref: Modern)								
Traditional	0.47	<0.001	0.19	<0.001	0.35	<0.001	0.41	<0.001
Density of TBA (Ref: Q1 - lowest)								
Q2 - low	1.10	0.504	0.70	0.294	0.88	0.442	1.32	0.164
Q3 - moderate	1.00	0.681	0.87	0.440	0.94	0.528	0.75	0.083
Q4 - high	1.05	0.581	0.37	0.001	0.60	0.010	0.46	<0.001
Q5 - highest	0.73	0.101	0.32	<0.001	0.50	0.001	0.38	<0.001
Mother's age at birth (Ref: 15-24 years)								
25-34	1.62	0.001	1.62	0.003	1.82	<0.001	1.74	<0.001
35-49	1.70	0.007	2.04	0.002	2.05	<0.001	2.20	<0.001
Mother's education (Ref:								

Primary school or less)								
Junior high school	1.42	0.006	2.10	<0.001	1.72	<0.001	1.52	0.001
Senior high school or more	2.22	<0.001	3.71	<0.001	2.50	<0.001	2.27	<0.001
Parity (Ref: 1)								
2	0.83	0.151	1.19	0.226	0.94	0.449	0.76	0.048
3	0.68	0.022	0.93	0.497	0.76	0.089	0.62	0.001
≥4	0.55	0.002	0.86	0.329	0.60	0.004	0.47	<0.001
Economic state (Ref: Poorest (Q1))								
Poor (Q2)	1.70	<0.001	1.01	0.647	1.60	<0.001	1.31	0.071
Middle (Q3)	1.83	<0.001	1.34	0.090	1.88	<0.001	1.51	0.005
Rich (Q4)	2.77	<0.001	1.58	0.042	2.65	<0.001	1.96	<0.001
Richest (Q5)	4.19	<0.001	2.11	0.012	3.91	<0.001	2.92	<0.001
Residence type (Ref: Rural)								
Urban	1.43	0.012	3.47	<0.001	1.93	<0.001	2.20	<0.001
**REGION: KALIMANTAN**								
Family structure (Ref: Nuclear)								
Extended	1.25	0.249	1.14	0.476	0.98	0.654	0.90	0.461
Composite Cultural barrier variable (Ref: Modern)								
Traditional	0.34	<0.001	0.15	<0.001	0.23	<0.001	0.61	0.056
Density of TBA (Ref: Q1 - lowest)								
Q2 - low	0.65	0.249	0.56	0.217	0.77	0.326	1.23	0.433
Q3 - moderate	0.61	0.149	0.87	0.576	0.77	0.340	1.34	0.390
Q4 - high	0.51	0.097	0.58	0.192	0.49	0.024	0.62	0.169
Q5 - highest	0.26	0.005	0.26	0.004	0.30	0.001	0.45	0.076
Mother’s age at birth (Ref: 15-24 years)								
25-34	1.37	0.073	2.24	0.003	1.67	0.024	1.62	0.055
35-49	1.25	0.323	4.28	<0.001	2.02	0.022	1.69	0.097
Mother's education (Ref: Primary school or less)								
Junior high school	1.99	0.008	2.06	0.007	2.18	0.004	1.92	0.008
Senior high school or more	2.40	<0.001	2.11	0.009	2.25	0.001	2.28	<0.001
Parity (Ref: 1)								
2	0.82	0.265	0.43	0.003	0.55	0.012	0.65	0.098
3	0.71	0.131	0.43	0.006	0.56	0.039	0.53	0.044
≥4	0.68	0.186	0.19	<0.001	0.46	0.024	0.71	0.229
Economic state (Ref: Poorest (Q1))								
Poor (Q2)	0.80	0.353	1.05	0.583	0.82	0.333	1.07	0.574
Middle (Q3)	1.13	0.480	1.30	0.188	1.17	0.383	1.37	0.292
Rich (Q4)	1.24	0.361	1.72	0.055	1.55	0.076	2.81	0.008
Richest (Q5)	2.05	0.058	3.11	0.008	2.90	0.002	4.63	<0.001
Residence type (Ref: Rural)								
Urban	1.24	0.319	2.13	0.069	1.76	0.097	2.41	0.060
**REGION: SULAWESI**								
Family structure (Ref: Nuclear)								
Extended	1.25	0.249	1.14	0.476	0.98	0.654	0.90	0.461
Composite Cultural barrier variable (Ref: Modern)								
Traditional	0.34	<0.001	0.15	<0.001	0.23	<0.001	0.61	0.056
Density of TBA (Ref: Q1 - lowest)								
Q2 - low	0.65	0.249	0.56	0.217	0.77	0.326	1.23	0.433

Q3 - moderate	0.61	0.149	0.87	0.576	0.77	0.340	1.34	0.390
Q4 - high	0.51	0.097	0.58	0.192	0.49	0.024	0.62	0.169
Q5 - highest	0.26	0.005	0.26	0.004	0.30	0.001	0.45	0.076
Mother's age at birth (Ref: 15-24 years)								
25-34	1.37	0.073	2.24	0.003	1.67	0.024	1.62	0.055
35-49	1.25	0.323	4.28	<0.001	2.02	0.022	1.69	0.097
Mother's education (Ref: Primary school or less)								
Junior high school	1.99	0.008	2.06	0.007	2.18	0.004	1.92	0.008
Senior high school or more	2.40	<0.001	2.11	0.009	2.25	0.001	2.28	<0.001
Parity (Ref: 1)								
2	0.82	0.265	0.43	0.003	0.55	0.012	0.65	0.098
3	0.71	0.131	0.43	0.006	0.56	0.039	0.53	0.044
≥4	0.68	0.186	0.19	<0.001	0.46	0.024	0.71	0.229
Economic state (Ref: Poorest (Q1))								
Poor (Q2)	0.80	0.353	1.05	0.583	0.82	0.333	1.07	0.574
Middle (Q3)	1.13	0.480	1.30	0.188	1.17	0.383	1.37	0.292
Rich (Q4)	1.24	0.361	1.72	0.055	1.55	0.076	2.81	0.008
Richest (Q5)	2.05	0.058	3.11	0.008	2.90	0.002	4.63	<0.001
Residence type (Ref: Rural)								
Urban	1.24	0.319	2.13	0.069	1.76	0.097	2.41	0.060
**REGION: EAST INDONESIA**								
Family structure (Ref: Nuclear)								
Extended	1.16	0.345	1.60	0.016	1.47	0.044	1.33	0.186
Composite Cultural barrier variable (Ref: Modern)								
Traditional	0.46	<0.001	0.20	<0.001	0.25	<0.001	0.31	<0.001
Density of TBA (Ref: Q1 - lowest)								
Q2 - low	0.55	0.091	1.34	0.498	0.68	0.280	0.87	0.531
Q3 - moderate	1.65	0.214	1.03	0.649	1.20	0.445	0.93	0.581
Q4 - high	1.61	0.192	0.67	0.251	0.86	0.482	1.08	0.604
Q5 - highest	0.78	0.267	0.31	0.002	0.44	0.022	0.51	0.087
Mother's age at birth (Ref: 15-24 years)								
25-34	1.54	0.058	1.81	0.008	1.78	0.008	1.71	0.043
35-49	1.45	0.166	1.70	0.094	1.86	0.032	1.61	0.103
Mother's education (Ref: Primary school or less)								
Junior high school	1.39	0.078	1.85	0.005	1.64	0.013	1.55	0.093
Senior high school or more	2.01	0.003	3.34	<0.001	2.60	<0.001	1.96	0.002
Parity (Ref: 1)								
2	0.53	0.016	0.64	0.097	0.61	0.034	0.61	0.023
3	0.44	0.004	0.54	0.024	0.56	0.018	0.51	0.007
≥4	0.48	0.036	0.52	0.035	0.45	0.003	0.38	0.001
Economic state (Ref: Poorest (Q1))								
Poor (Q2)	1.08	0.524	0.98	0.660	1.03	0.622	0.97	0.617
Middle (Q3)	1.53	0.076	1.27	0.234	1.49	0.081	1.43	0.127
Rich (Q4)	1.41	0.210	1.43	0.163	1.56	0.094	1.66	0.074
Richest (Q5)	1.53	0.160	2.48	0.049	2.20	0.031	1.77	0.096
Residence type (Ref: Rural)								
Urban	1.38	0.126	1.64	0.078	1.46	0.081	2.28	0.002
